# Regulating Al_2_O_3_/PAN/PEG Nanofiber Membranes with Suitable Phase Change Thermoregulation Features

**DOI:** 10.3390/nano13162313

**Published:** 2023-08-12

**Authors:** Leping Huang, Ying Chen, Zhaobao Xu, Cui He, Youmu Li, Jinchao Zhao, Youhong Tang

**Affiliations:** 1 College of Materials Science and Engineering, Wuhan Textile University, Wuhan 430200, China; lphuang@wtu.edu.cn (L.H.); 2215043051@mail.wtu.edu.cn (Y.C.); 2215383110@mail.wtu.edu.cn (Y.L.); 2 Hubei Provincial Engineering Laboratory for Clean Production and High Value Utilization of Bio-Based Textile Materials, Wuhan Textile University, Wuhan 430200, China; zhaobao.xu@lianhetech.com (Z.X.); hecui10127@outlook.com (C.H.); 3 Flinders Institute for NanoScale Science and Technology, College of Science and Engineering, Flinders University, Adelaide, SA 5042, Australia

**Keywords:** coaxial electrospinning, nanofiber membranes, core-shell structure, phase change, temperature-regulating

## Abstract

To address the thermal comfort needs of the human body, the development of personal thermal management textile is critical. Phase change materials (PCMs) have a wide range of applications in thermal management due to their large thermal storage capacity and their isothermal properties during phase change. However, their inherent low thermal conductivity and susceptibility to leakage severely limit their application range. In this study, polyethylene glycol (PEG) was used as the PCM and polyacrylonitrile (PAN) as the polymer backbone, and the thermal conductivity was increased by adding spherical nano-alumina (Al_2_O_3_). Utilizing coaxial electrospinning technology, phase-change thermoregulated nanofiber membranes with a core-shell structure were created. The study demonstrates that the membranes perform best in terms of thermal responsiveness and thermoregulation when 5% Al_2_O_3_ is added. The prepared nanofiber membranes have a melting enthalpy of 60.05 J·g^−1^ and retain a high enthalpy after 50 cycles of cold and heat, thus withstanding sudden changes in ambient temperature well. Additionally, the nanofiber membranes have excellent air permeability and high moisture permeability, which can increase wearer comfort. As a result, the constructed coaxial phase change thermoregulated nanofiber membranes can be used as a promising textile for personal thermal management.

## 1. Introduction

The state of one’s health can be impacted by thermal comfort. By protecting the body from the outside elements and insulating the air from the human body, clothing is essential to ensuring that the body’s internal temperature remains consistent [1,2,3,4]. Through two major principles, advanced textiles for personal thermal management (PTM) are intended to increase body heat production and decrease heat loss from the body. In reaction to changes in the external environment and the body’s internal temperature, these intelligent thermoregulation textiles may actively regulate the temperature of the microenvironment within the body, improving human comfort [5,6,7,8,9]. It is more accurate and energy efficient than traditional indoor thermal regulation methods.

Phase change materials (PCMs) are substances that absorb or release heat from the environment through their phase change within a specific temperature range [10,11,12]. PCMs have the capacity to swiftly absorb, diffuse, and release heat, enabling bi-directional temperature regulation [13,14,15,16,17,18]. PCM-based textiles exhibit a cushioning effect on the environment and can keep the body in a comfortable temperature range [19,20,21,22,23]. Polyethylene glycol (PEG) is a typical polymeric PCM. PEGs of various molecular weights can be combined by heating to the necessary phase transition temperature to satisfy the body’s comfort requirements. PEG plays a significant role in temperature-regulating textiles because it has the properties of structural rule, strong crystallinity, good biocompatibility, and high latent heat of phase change. 

However, PCM in mixed fibers can be easily removed through cleaning, wiping, or abrasion, and there is also the issue of leaking during the phase change process, which results in poor material stability and a short service life [24,25,26]. To tackle this challenge, encapsulation technology for PCM has become the focus of the study. Container packaging, microencapsulation, porous adsorption, electrostatic spinning, and other technologies are commonly used for encapsulation [27,28,29]. Electrostatic spinning is a simple and effective new technology for processing micro and nanofibers that has a wide range of applications in biomedical materials, filtration protection, catalysis, energy, optoelectronics, food engineering, cosmetics, and other areas. It has the benefits of variable fiber composition, fine customizable structure, big specific surface area, high porosity, and good flexibility of the manufactured fibers, which may suit human comfort requirements [30,31,32]. Coaxial electrospinning has shown its obvious advantages, with concentrically arranged spinnerets connected to separate channels of different solutions allowing the direct production of fibers with a core-shell structure. PEG can be directly encapsulated in the core layer of the polymer using coaxial electrospinning to solve leakage problems [33,34]. PEG has the disadvantage of poor thermal conductivity, which limits its heat transfer properties [35]. However, the thermal conductivity of PCM is important, as this determines the rate at which the PCM reaches the solid-liquid transition and also affects the efficiency of energy storage and release [36,37]. This problem can be solved by adding nanoscale thermally conductive fillers such as graphene, copper particles, alumina, etc. Among them, nano-alumina has excellent properties like high thermal conductivity, good insulation, great hardness, high-temperature resistance, corrosion resistance, wear resistance, excellent flowability, and controllable particle size distribution. In this study, spherical nano-alumina (Al_2_O_3_) is added to PCM based on PEG to improve the thermal conductivity of PCM. High latent heat, fast heat transfer, good circulation stability, and the ability to meet human comfort needs are the directions for the development of phase change thermoregulated textiles. To fulfill the needs of the human body, we combined PEGs with different molecular weights and regulated their phase change temperatures to the range of 33–38 °C. The composite PEG was then used as the core layer, polyacrylonitrile (PAN) was used as the shell layer, and Al_2_O_3_ was added to the shell layer to prepare the core-shell structure of Al_2_O_3_/PAN/PEG phase change thermoregulation nanofiber membranes. The results show that the nanofiber membranes prepared in this research are promising for PTM.

## 2. Materials and Methods

### 2.1. Materials

Polyacrylonitrile (PAN, average Mw = 150,000) was purchased from Shanghai Macklin Biochemical Technology Co., Ltd., China. N, N-dimethylformamide (DMF) and PEG1500 were purchased from Sinopharm Group Co., Ltd., Shanghai, China. PEG1000 was purchased from Xi’an Tianzheng Co., Ltd., China. Spherical nano-alumina (Al_2_O_3_, average particle size of 30 nm) was purchased from Beijing Deke Daojin Science and Pharmaceutical Technology Co., Ltd., China. All these materials were used without further processing and purification.

### 2.2. Compounding of PEG

The phase-change temperature of pure PEG with different molecular weights was characterized by a differential scanning thermal analyzer (DSC, 200F3, NETZSCH, Selb, Germany), and then the optimal mixture ratio was determined by using the SchrÖder Equation (1):

(1) $$T=\frac{1}{\frac{1}{T_{f}}-\frac{R\ln X_{A}}{\Delta_{s}^{l}H_{A}}}$$

where *X_A_* is the mole fraction of pure solid compound *A* in the composite phase change material, $\Delta_{s}^{l}$*H_A_* is the phase change enthalpy of pure solid compound *A*, $T_{f}$ is the phase change temperature of pure solid compound *A*, *T* is the phase change temperature of the composite PCM containing solid compound *A*, and *R* is the gas constant. A hot melting process was used to combine PEG with different molecular weights at the theoretical ideal ratio. The DSC test confirmed the theoretical ratio’s rationality. The composite PEG with a phase change temperature of 33–38 °C was chosen as the core spinning solution.

### 2.3. Preparation of Shell-Spinning Solution 

A certain mass of Al_2_O_3_ was dispersed in DMF and sonicated for 1 h. Then, PAN was added and agitated for 2 h at a constant temperature of 70 °C to generate an Al_2_O_3_/PAN shell-spinning solution containing 10% PAN. In the experiment, the concentrations of regulated Al_2_O_3_ were 1%, 3%, 5%, 7%, and 9%, respectively. 

### 2.4. Preparation of Al_2_O_3_/PAN/PEG Nanofiber Membranes

**Figure 1:**
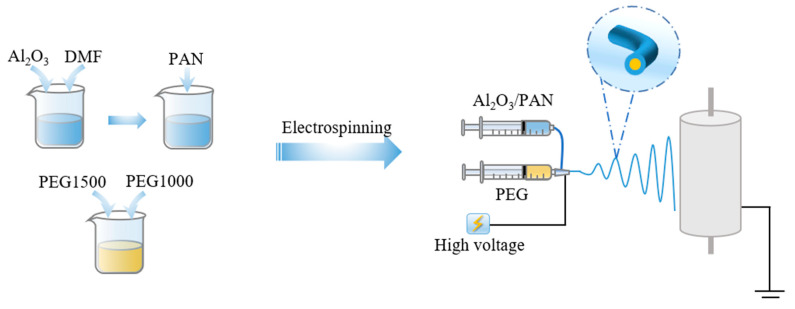
Schematic diagram of the preparation of phase change thermoregulated nanofiber membranes by coaxial electrospinning.

The prepared solution was transferred into a 20 mL disposable syringe, and electrostatic spinning was performed with a coaxial needle with an inner diameter of 20 G and an outer diameter of 14 G. Electrostatic spinning was performed at an applied voltage of 16 kV, a spinning distance of 10 cm, a core layer advancement speed of 0.015 mm·min^−1^, a shell layer advancement speed of 0.08 mm·min^−1^, and a drum receiver speed of 100 r·min^−1^ for 2 h. Finally, the obtained nanofiber membranes were vacuum dried at room temperature for 24 h and stored in a desiccator for use. According to the change of Al_2_O_3_ concentration, the resulting phase change thermoregulation nanofiber membranes were named 1% Al_2_O_3_/PAN/PEG, 3% Al_2_O_3_/PAN/PEG, 5% Al_2_O_3_/PAN/PEG, 7% Al_2_O_3_/PAN/PEG, and 9% Al_2_O_3_/PAN/PEG, respectively. The schematic illustrations are shown in Figure 1. 

### 2.5. Characterization of Al_2_O_3_/PAN/PEG Nanofiber Membranes 

The surface morphology and structure features were determined using scanning electron microscopy (SEM, JSM-IT300, JEOL, Akishima, Japan). The thermal stability of the phase change nanofiber membranes were characterized by thermogravimetric analysis (STA 2500 Regulus, NETZSCH, Germany) with N_2_ as the protective gas, the temperature range of 30–800 °C, and the ramp-up rate of 10 °C·min^−1^.The phase change behavior of the compounded PCMs and the phase change nanofiber membranes were characterized by differential scanning calorimetry (DSC, 200F3, NETZSCH, Selb, Germany) with N_2_ as the protective gas, the temperature range of 0–100 °C, and the ramp-up/down rate of 10 °C·min^−1^. The membrane thermal regulating properties of the phase change nanofiber membranes were characterized using infrared thermography (FLIR, E8, TELEDYNE, Boston, MA, USA) with a sample size of 2 cm × 2 cm × 0. 01 cm, and the temperature of the hot bench was increased from room temperature to 50 °C. The air permeability of the phase change nanofiber membranes were characterized by a fabric air permeability tester (YG(B) 461G, Wenzhou Da Rong Spinning Instrument, Wenzhou, China). The moisture permeability of the phase change nanofiber membranes was tested by the evaporation method using a computerized fabric moisture meter (YG601H, Ningbo Textile Instrument Factory, Ningbo, China) at 38 °C, humidity of 50%, and gas flow rate of 0.3 m·s^−1^. The sample is weighed after being balanced for 0.5 h in the test chamber and then weighed again after 1 h of testing. The water vapor transmission rate (WVTR) is calculated according to Formula (2):

(2) $$WVTR=\frac{W_{1}-W_{2}}{S}\times 24$$

where *S* is the effective test area (where *S* is 2.826×10^−3^ m^2^), *W*_1_ is the first weighing weight, and *W*_2_ is the second weighing weight [38].

## 3. Results and Discussion

### 3.1. Ratio of Complex PEG

**Figure 2:**
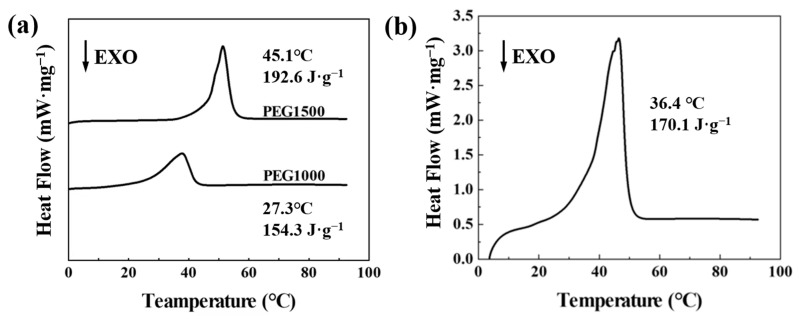
(**a**) DSC curves for PEG1500 and PEG1000. (**b**) DSC curve for PEG compounded at a 1:9 molar ratio of PEG1500 and PEG1000.

From the standpoint of the actual thermal management application, the phase transition behavior is of utmost significance. The DSC test results for PEG1500 and PEG1000’s phase change temperature and thermal energy storage capacity are depicted in Figure 2a. The actual phase change temperature of PEG1500 and PEG1000 are 45.1 °C and 27.3 °C, respectively. The phase change enthalpy for PEG1500 and PEG1000 are 192.6 J·g^−1^ and 154.3 J·g^−1^. The phase change temperatures of both materials do not meet the human comfort requirements. According to the SchrÖder equation, the theoretical phase change temperature of the composite PEG is 35 °C when the molar fraction ratio of PEG1500 to PEG1000 is 1:9 (i.e., 1:6 by mass). After DSC testing, the phase change temperature of the aforesaid blended PEG is 36.4 °C, and the phase change enthalpy is 170.1 J·g^−1^, which meets the human comfort requirements, as shown in Figure 2b. Therefore, PEG1500:PEG1000 = 1:9 was used as the core layer solution to prepare the phase change thermoregulated nanofiber membranes.

### 3.2. The Structure and Morphology of Al_2_O_3_/PAN/PEG

**Figure 3:**
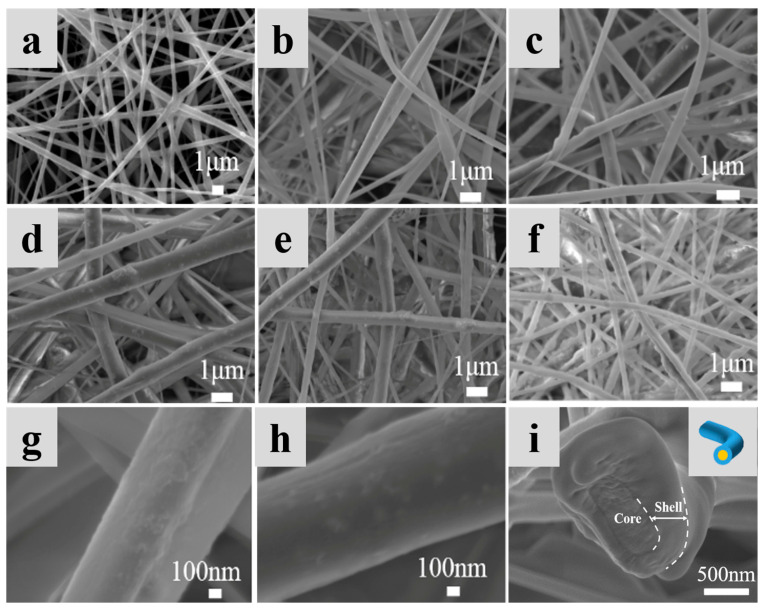
SEM images of nanofiber membranes containing different concentrations of Al_2_O_3_: (**a**) PAN/PEG, (**b**) 1% Al_2_O_3_/PAN/PEG, (**c**) 3% Al_2_O_3_/PAN/PEG, (**d**) 5% Al_2_O_3_/PAN/PEG, (**e**) 7% Al_2_O_3_/PAN/PEG and (**f**) 9% Al_2_O_3_/PAN/PEG. (**g**,**h**) are high-magnification images of (**b**,**d**), respectively. (**i**) is a cross-sectional view of 5% Al_2_O_3_/PAN/PEG, and the inset is a schematic diagram of a core-shell structured nanofiber.

To ensure that the PCM can be used properly above the melting point and does not leak, we have prepared phase change nanofiber membranes with a core-shell structure by encapsulating PEG in the fibers using coaxial electrospinning technology. Figure 3a–f displays SEM images of nanofiber membranes prepared by adding Al_2_O_3_ at 0%, 1%, 3%, 5%, 7%, and 9% concentrations to the shell layer, respectively. Randomly oriented cylindrical fibers can be observed. 

**Figure 4:**
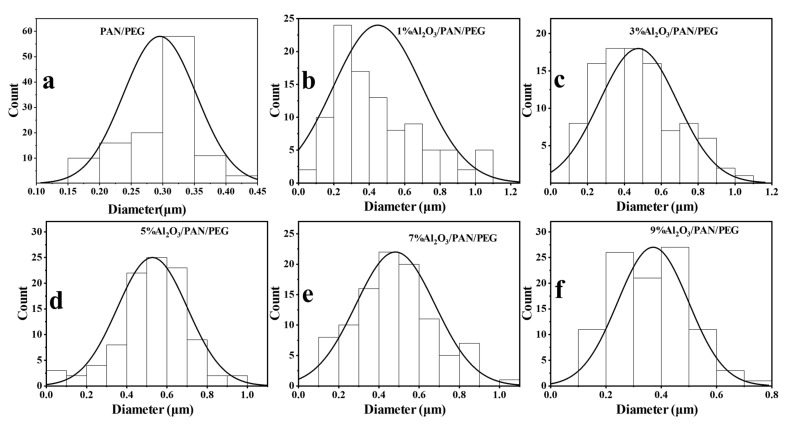
Distribution of diameters of nanofiber membranes containing different concentrations of Al_2_O_3_: (**a**) PAN/PEG, (**b**) 1% Al_2_O_3_/PAN/PEG, (**c**) 3% Al_2_O_3_/PAN/PEG, (**d**) 5% Al_2_O_3_/PAN/PEG, (**e**) 7% Al_2_O_3_/PAN/PEG, and (**f**) 9% Al_2_O_3_/PAN/PEG.

The PAN/PEG fibers modified without the addition of Al_2_O_3_ have the smallest diameter. As the concentration of Al_2_O_3_ increases, it can be observed that the surface of the fibers gradually becomes rougher, the fibers become progressively thicker, and the diameter distribution becomes wider. The fibers are thickest when 5% Al_2_O_3_ is added, with an average diameter of 528 ± 175 nm. This was due to the increase in surface loading of the fibers as a result of the increase in Al_2_O_3_ concentration in the shell layer. As the concentration of Al_2_O_3_ continues to increase, the average diameter of the fibers gradually decreases, indicating that the amount of Al_2_O_3_ loaded on the fiber surface has reached saturation. At the same time, the addition of Al_2_O_3_ increases the viscosity of the spinning solution. The greater the viscosity, the more difficult it is to stretch the fibers under the electric field forces, the more difficult electrostatic spinning will be, and the larger the diameter of the fibers will be. In addition, the increased electrical conductivity of the spinning solution enhances the stretching effect of the jet, leading to a reduction in the diameter of the fibers [39]. Based on SEM images, the diameter of the fibers and the variation of the fibers can be quantitatively assessed. The direct distribution of the various nanofiber membranes is shown in Figure 4, and the average diameter is shown in Table 1. The phenomenon of fibers adhering to each other can also be observed, which is caused by incomplete evaporation of the solvent from adjacent fibers. Figure 3g,h shows that as the Al_2_O_3_ concentration increases from 0% to 5%, the distribution of Al_2_O_3_ on the fiber surface increases and becomes more homogeneous. The higher the percentage of Al_2_O_3_ in the fiber, the better the thermal conductivity. Figure 3i shows a cross-sectional view of the 5% Al_2_O_3_/PAN/PEG fiber, where a distinct core-shell structure can be observed, indicating that the addition of Al_2_O_3_ barely affects the construction of the core-shell structure in the fiber and that the PEG well encapsulated.

### 3.3. Thermal Stability Analysis

**Figure 5:**
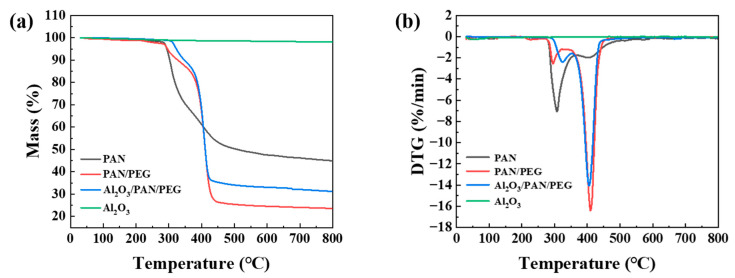
TG/DTG test diagram of PAN, PAN/PEG, 5% Al_2_O_3_/PAN/PEG: (**a**) TG and (**b**) DTG.

An important factor affecting the working temperature range of composites is the thermal stability of the material. To investigate the effect of Al_2_O_3_ modification on the thermal stability of nanofiber membranes, thermogravimetric analysis was carried out on Al_2_O_3_, PAN, PAN/PEG, and 5% Al_2_O_3_/PAN/PEG. Figure 5 shows the TG and DTG curves, respectively. The thermogravimetric analysis shows that the degradation trends of Al_2_O_3_/PAN/PEG and PAN/PEG are similar. A two-step weight loss process occurs during the warming process. At 300 °C, the weight loss components are dominated by PAN and water. The rate of degradation is greatest between 350 °C and 450 °C, with the main weight loss being the PEG fraction. At 50% weight loss, PAN is at 550 °C, while PAN/PEG and Al_2_O_3_/PAN/PEG are at 410 °C, indicating that PAN/PEG and Al_2_O_3_/PAN/PEG have better thermal stability than PAN. The decomposition temperature of Al_2_O_3_/PAN/PEG is 324 °C. After 550 °C, the curves are largely stable. The addition of Al_2_O_3_ increased the residue from 24% to 31% compared to PAN/PEG after curves stabilization. These phenomena suggest that highly stable layers of Al_2_O_3_ nanoparticles are involved in the carbon formation process, resulting in a denser and more stable carbon layer that boosts the internal matrix’s resistance to thermal degradation.

### 3.4. Analysis of Thermal Conductivity and Storage Performance

**Figure 6:**
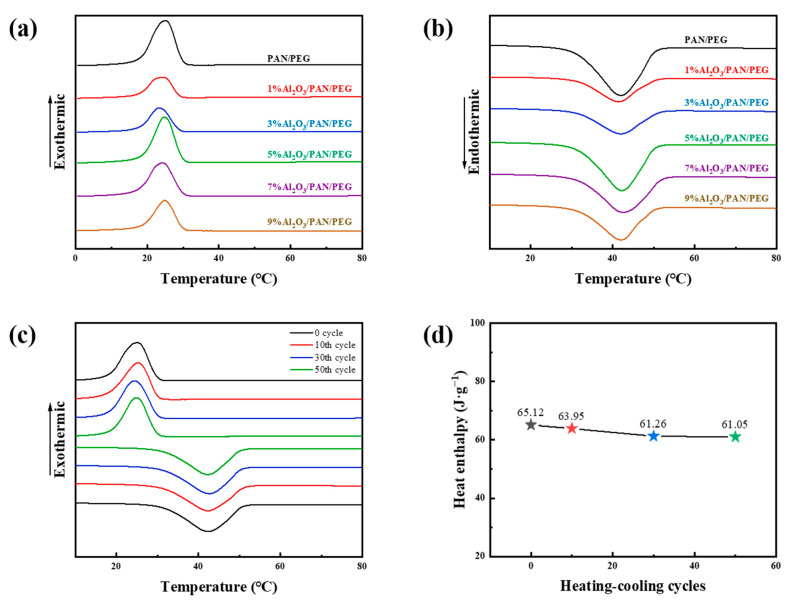
DSC curves of fibrous membranes with varying Al_2_O_3_ concentrations during the (**a**) cooling and (**b**) melting processes. 5% Al_2_O_3_/PAN/PEG after heating and cooling cycles (**c**) DSC curves and (**d**) encapsulation efficiency plot.

The latent heat properties of PCMs are important for assessing their thermal storage capacity. The phase transition temperature and thermal storage capacity of phase change nanofiber membranes with different Al_2_O_3_ concentrations were investigated by differential scanning calorimetry. As shown in Figure 6, nanofiber membranes with different Al_2_O_3_ concentrations exhibit similar characteristic profiles during heating and cooling, with both heat absorption and exothermic peaks, which are attributed to the reversible phase change. The exothermic peak is caused by nucleation crystallization, indicating a liquid–solid phase transition. The heat absorption peak corresponds to a phase change from an ordered crystalline phase to a disordered amorphous state and represents a solid–liquid phase transition. The latent heats of melting and cooling represent the energy released and absorbed during the heating and cooling processes and can visualize the ability of the PCM to thermally regulate in both directions. The DSC curves for these phase change nanofiber membranes all exhibit appropriate enthalpies and melting/cooling temperatures in the human comfort temperature range.

As shown in Table 2, the melting point (*T_m_*) of PAN/PEG was 31.8 °C, close to human skin temperature, and the melting enthalpy (Δ*H_m_*) of PAN/PEG was 70.38 J·g^−1^. However, the phase change nanofiber membranes prepared with the addition of Al_2_O_3_ had lower enthalpy of melting and cooling compared to PAN/PEG due to the fact that only PEG was responsible for the composite fiber in a certain temperature range. The introduction of Al_2_O_3_ reduces the mass ratio of PEG. In addition, PEG has the disadvantage of being poorly spinnable, which affects the amount of PEG encapsulated in the fiber, while increasing the PEG content helps to enhance the latent heat of phase change of the PCM. It is worth noting that the peak melt temperature (*T_mp_*) shifts towards a lower temperature with the addition of Al_2_O_3_. This is because the Al_2_O_3_ addition makes the fibrous membrane more thermally sensitive, which makes it simpler for the PEG to reach the phase change. After modification with Al_2_O_3_, the enthalpy of 5% Al_2_O_3_/PAN/PEG is the highest at 60.05 J·g^−1^. The thickest fiber and saturation of the surface Al_2_O_3_ at this point are consistent with the morphological analysis above. Compared to PAN/PEG, the cooling enthalpy (∆*H_c_*) for 5% Al_2_O_3_/PAN/PEG is lower, and the onset of cooling temperature (*T_co_*) is slightly higher. These are most likely owing to the inclusion of Al_2_O_3_, which improves heat transfer and results in a lower nucleation barrier for the PEG to form stable nuclei, hence boosting cooling temperature and decreasing cooling enthalpy. In summary, 5% Al_2_O_3_/PAN/PEG has high latent heat and thermal sensitivity. Furthermore, because of the appropriate *T_m_*, this material is a practical candidate for thermally regulated textiles.

As the temperature rises, the phase change material transforms from a solid to a liquid. In the actual application process, the phase change material transformed into a liquid state is easy to flow in the core layer, and multiple cycles of melting and solidifying will also cause the flow of phase change material in the core layer. If the phase change material is not fully coated, it will easily leak out from it and be lost in the process of use due to friction, washing, and other factors, thus affecting its subsequent thermoregulation performance. The thermal cycling incalculability of phase change nanofiber membranes affects the effectiveness and lifetime of the material. To investigate the encapsulation stability of phase change nanofiber membranes, heating/cooling cycles of 5% Al_2_O_3_/PAN/PEG were carried out in a constant temperature oven at 20 to 70 °C. The phase change properties of 5% Al_2_O_3_/PAN/PEG after 10, 20, 30, and 50 cycles were investigated by DSC. As can be seen in Figure 5c, the phase change temperatures of 5% Al_2_O_3_/PAN/PEG during heating and cooling remained almost constant over 50 cycles, and the melting and cooling peaks showed good overlap in position and intensity, indicating a stable and reversible phase change behavior of the nanofiber membranes. As can be seen in Figure 5d, the initial melting enthalpy of the nanofiber membranes was 65.12 J·g^−1^, which decreased to 61.05 J·g^−1^ after 50 cycles, only about 6.25% lower than the initial value. The enthalpy of crystallization changed from 60.21 J·g^−1^ to 57.78 J·g^−1^. These results suggest that a small amount of PEG may have been incompletely encapsulated after 50 cycles and leaked out of the phase change thermoregulation nanofiber membranes when it warmed up to a liquid state, which was lost during multiple accesses to the heating/cooling cycling test, leading to a decrease in the enthalpy of the nanofiber membranes. Together, the nanofiber membranes still had high latent heat after multiple thermal cycling tests. The Al_2_O_3_/PAN/PEG phase change thermoregulated nanofiber membranes have a high energy storage density, suitable phase change temperature, and stable thermal cycling, confirming their potential for PTM applications.

**Figure 7:**
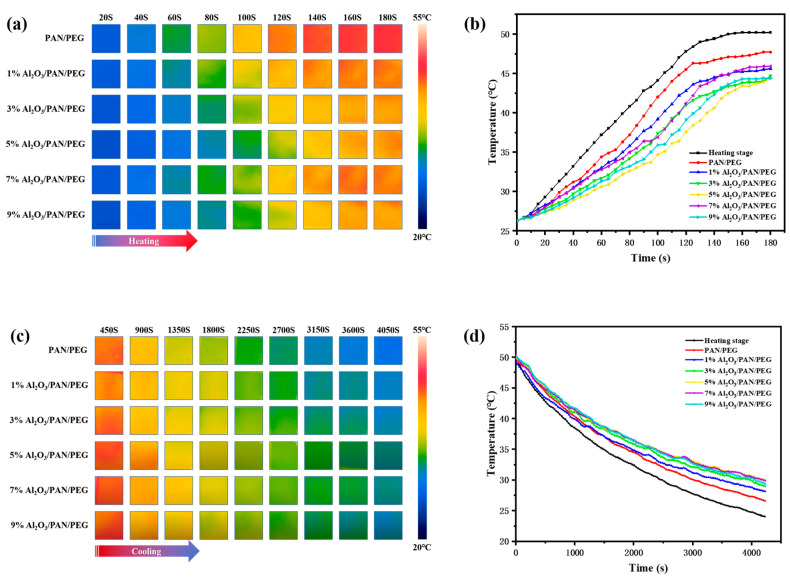
Representative thermal images of nanofiber membranes recorded by infrared thermography during (**a**) heating and (**c**) cooling process. Temperature evolution plots of the corresponding membranes in the (**b**) heating and (**d**) cooling processes.

To investigate the effect of Al_2_O_3_ on the thermal conductivity of phase change thermoregulated nanofiber membranes, an infrared thermographer connected to a computer was used to record in real time the temperature change of the nanofiber membranes on the thermal bench. The heating stage was first brought up to 50 °C from room temperature, and the temperature rise variations were recorded. When the temperature of all nanofiber membranes stabilized at 50 °C, the heating was turned off, and the cooling variations were recorded. Figure 7a,c shows the thermograms for different Al_2_O_3_ concentrations in the nanofiber membranes. All the nanofiber membranes remained at the same ambient temperature (26.3 °C) at 0 s after the heating stage started to heat up. The surface temperatures of the nanofiber membranes were similar after 20 s. However, the 5% Al_2_O_3_/PAN/PEG surface temperature was the lowest (27.3 °C). It is noteworthy that the difference in surface temperature starts to increase after 60 s, with the largest difference at 120 s. The 5% Al_2_O_3_/PAN/PEG (37.6 °C) is significantly lower than the PAN/PEG sample (45.5 °C), indicating that the addition of Al_2_O_3_ gives the nanofiber membranes excellent thermal conductivity and thus allows the phase change material to absorb more heat. The 5% Al_2_O_3_/PAN/PEG surface temperature was the lowest compared to the 7% Al_2_O_3_/PAN/PEG (42.4 °C) and 9% Al_2_O_3_/PAN/PEG (39.1 °C), indicating that the thermal conductivity was optimal and that the 5% Al_2_O_3_ was saturated in the fibers. Similarly, throughout the cooling process, a similar thermal response trend can be observed, with the surface temperature of the 5% Al_2_O_3_/PAN/PEG sample decreasing relatively slowly. The thermal images illustrate that 5% Al_2_O_3_/PAN/PEG has the best thermal regulation. To provide a clearer picture of the trend of the nanofiber membranes temperature change, the temperature was recorded every 5 s, resulting in the temperature change curves shown in Figure 7b,d. Compared to the heating stage, the nanofiber membranes exhibited slower temperature changes during the same heating and cooling phases due to the presence of the phase change material PEG, with the 5% Al_2_O_3_/PAN/PEG always having the smoothest temperature change profile. This is because the phase change material PEG in the Al_2_O_3_/PAN/PEG undergoes a solid-liquid phase change during heating, which absorbs heat and thus reduces the warming rate of the nanofiber membrane, and a liquid–solid phase change during cooling, which releases heat and thus slows down the cooling rate of the nanofiber membranes. These results show that the Al_2_O_3_-modified PEG/PAN with a shell layer has a significantly lower temperature change rate compared to the original nanofiber membranes due to the presence of Al_2_O_3_ nanoparticles within the fiber. This demonstrates that the PAN/PEG nanofiber membranes modified with Al_2_O_3_ nanoparticles have thermal storage and temperature regulation properties at this temperature. The best thermoregulation performance was achieved at a concentration of 5% Al_2_O_3_ in the shell layer of the modified nanofiber membranes. The thermal performance of nanofiber membranes can be adversely affected by either increasing or decreasing concentrations.

### 3.5. Analysis of Breathability and Moisture Permeability

**Figure 8:**
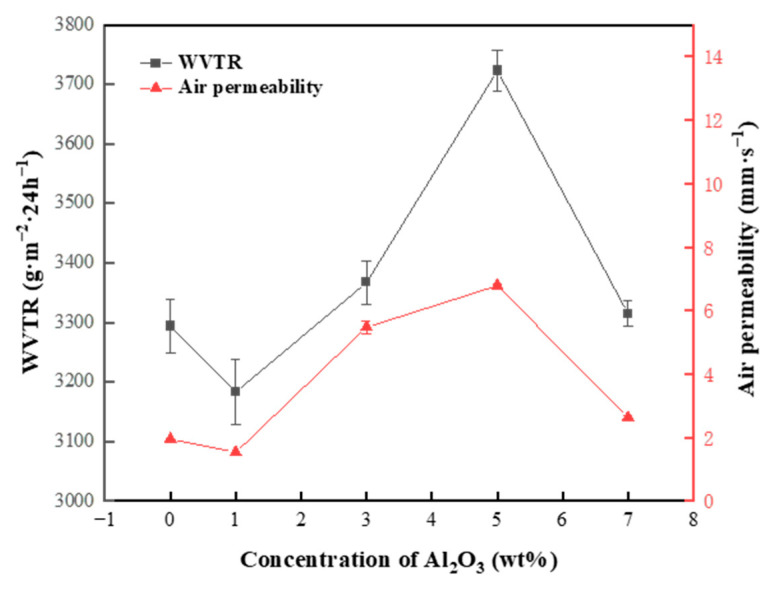
Air permeability and moisture permeability of nanofiber membranes with different Al_2_O_3_ concentrations.

The breathability and moisture permeability of the fabric together influences the comfort of human wear [40]. The breathability of different phase change nanofiber membranes was tested according to GB/T 5453, using the evaporation method (positive cup), as shown in Figure 8. The air permeability and moisture permeability curves of the nanofiber membranes showed a similar trend, with an increase in air permeability favoring an increase in moisture permeability. The WVTR of all the membranes was above 3000 g·m^−2^·24 h^−1^, with the highest WVTR of 3723 g·m^−2^·24 h^−1^at 5% Al_2_O_3_ because both PAN and PEG are hydrophilic materials where the hydrophilic groups help to increase the permeability of water vapor. When the concentration of Al_2_O_3_ is 5%, the fibers in the phase change membrane are the thickest, therefore increasing the gap between the fibers, which is conducive to the passage of water vapor. As Al_2_O_3_ increases, solvent evaporation is incomplete, bonding between fibers occurs, porosity decreases, and, therefore, water vapor permeability decreases. When the concentration of Al_2_O_3_ is 5%, the nanofiber membranes have the best moisture permeability and appropriate breathability, providing a certain degree of comfort for human wear.

## 4. Conclusions

In summary, in this study, Al_2_O_3_/PAN/PEG phase change thermoregulated nanofiber membranes with a core-shell structure were prepared using a coaxial electrospinning method by adding spherical nano Al_2_O_3_ to the shell layer PAN and increasing the response rate of the core layer phase change material PEG. When the molar fraction ratio of PEG1500 to PEG1000 is 1:9, the phase change temperature of the nanofiber membranes was adjustable between 33 °C and 38 °C, which meets the needs of human comfort. The best overall performance of the resulting nanofiber membranes was achieved at a 5% concentration of added Al_2_O_3_, with a uniform fiber diameter distribution, a latent heat of melting of 60.05 J·g^−1^, an onset of phase change temperature of 31.9 °C, and WVTR of 3723 g·m^−2^·24 h^−1^, in line with human comfort requirements. During heating, the heating rate of Al_2_O_3_/PAN/PEG was slower compared to that of the nanofiber membranes without Al_2_O_3_ modification, indicating that the addition of Al_2_O_3_ improved the heat transfer efficiency of the nanofiber membranes. In addition, the nanofiber membranes still had good thermal regulation after 50 cycles of heat and cold. This is due to the effective encapsulation of the core layer of PEG by the coaxial electrospinning technique. The prepared phase change thermoregulation nanofiber membranes have the comprehensive performance of fast response, reversible phase change, good cycling stability, high moisture permeability, and suitable air permeability, which is expected to be used for PTM textiles.

## Figures and Tables

**Table 1:** Nanofiber diameter data for different Al_2_O_3_ concentrations.

Sample	Average Diameter/nm	Standard Deviation/nm
PAN/PEG	300	150
1%Al_2_O_3_/PAN/PEG	448	253
3%Al_2_O_3_/PAN/PEG	476	211
5%Al_2_O_3_/PAN/PEG	528	175
7%Al_2_O_3_/PAN/PEG	482	197
9%Al_2_O_3_/PAN/PEG	371	128

**Table 2:** Enthalpy of nanofiber membranes with different Al_2_O_3_ concentrations.

Sample	Melting			Cooling		
	*T_mo_*/°C	*T_mp_*/°C	∆*H_m_*/J·g^−1^	*T_co_*/°C	*T_cp_*/°C	∆*H_c_*/J·g^−1^
PAN/PEG	31.8	42.3	70.38	18.4	25.1	−69.12
1%Al_2_O_3_/PAN/PEG	31.5	41.4	30.48	18.0	24.6	−27.64
3%Al_2_O_3_/PAN/PEG	31.6	41.3	32.62	18.2	23.3	−30.36
5%Al_2_O_3_/PAN/PEG	31.9	41.1	60.05	19.1	24.8	−54.78
7%Al_2_O_3_/PAN/PEG	32.3	41.2	52.01	18.0	24.3	−47.06
9%Al_2_O_3_/PAN/PEG	32.2	41.6	41.34	19.2	25.0	−36.39

Note: *T_m_* is the melt temperature on the ramp-up scan curve; *T_mo_* is the melt onset temperature; *T_mp_* is the peak melt temperature; ∆*H_m_* is the melting enthalpy; T_c_ is the cooling temperature on the ramp-down curve; *T_co_* is the cooling onset temperature; *T_cp_* is the peak cooling temperature; and ∆*H_c_* is the cooling enthalpy.

## Data Availability

The data are available from the corresponding authors on reasonable request.
